# CEPB dataset: a photorealistic dataset to foster the research on bin picking in cluttered environments

**DOI:** 10.3389/frobt.2024.1222465

**Published:** 2024-05-16

**Authors:** Paolo Tripicchio, Salvatore D’Avella, Carlo Alberto Avizzano

**Affiliations:** Department of Excellence in Robotics and AI, Institute of Mechanical Intelligence, Scuola Superiore Sant’anna, Pisa, Italy

**Keywords:** dataset, grasping, manipulation, bin-picking, machine vision

## Abstract

Several datasets have been proposed in the literature, focusing on object detection and pose estimation. The majority of them are interested in recognizing isolated objects or the pose of objects in well-organized scenarios. This work introduces a novel dataset that aims to stress vision algorithms in the difficult task of object detection and pose estimation in highly cluttered scenes concerning the specific case of bin picking for the Cluttered Environment Picking Benchmark (CEPB). The dataset provides about 1.5M virtually generated photo-realistic images (RGB + depth + normals + segmentation) of 50K annotated cluttered scenes mixing rigid, soft, and deformable objects of varying sizes used in existing robotic picking benchmarks together with their 3D models (40 objects). Such images include three different camera positions, three light conditions, and multiple High Dynamic Range Imaging (HDRI) maps for domain randomization purposes. The annotations contain the 2D and 3D bounding boxes of the involved objects, the centroids’ poses (translation + quaternion), and the visibility percentage of the objects’ surfaces. Nearly 10K separated object images are presented to perform simple tests and compare them with more complex cluttered scenarios tests. A baseline performed with the DOPE neural network is reported to highlight the challenges introduced by the novel dataset.

## 1 Introduction

Robotic manipulation is a complex task typically accomplished by employing a robotic manipulator and a vision system (DAvella et al., 2023a). In most of the existing solutions, the working pipeline consists of visualizing the scene, understanding the objects’ displacements, detecting the target, synthesizing a grasping pose to pick the object, planning a collision-free trajectory, and then grabbing the item. Therefore, vision holds an important role in the grasping process. The recent trend is to employ deep neural networks (DNNs), especially in the form of the popular Convolution Neural Networks, at least in one of the phases of the vision pipeline (Zhao et al., 2019): object recognition, object detection, or grasping point localization. Although DNNs are appealing since they can learn more complex features without manual design with respect to traditional local feature descriptors and shallow architectures, they need a large amount of training data to infer such features. Furthermore, supervised learning requires additional time to label training and testing data that usually consists of images for this kind of problem.

In the recent past, research on manipulation was mainly performed on flat surfaces like tabletops with well-distinct and separated objects (DAvella et al., 2022). Practical industrial scenarios like warehouse automation require working in more stringent conditions, facing the problems of tight space and clutter. Picking objects from a bin, also known as bin picking, is an evident use case and an important logistics application. Therefore, developing an algorithm able to get accurate pose estimation is fundamental for solving such kinds of tasks typical of flexible warehouses and manufacturing facilities in which the poses of the objects are unknown *a priori*. However, clutter complicates the detection of the targets due to multiple occlusions. Nowadays, scenes containing just the target item can be easily addressed by most of the existing solutions. However, adding even a few objects presenting similar colors or visual features can lead such techniques to fail (Hodan et al., 2018). Increasing the amount of clutter in the scene drastically changes the results that state-of-the-art pose estimation algorithms can achieve. Therefore, an important goal in this ongoing industrial revolution is to make such algorithms robust to clutter to increase the flexibility of the next-generation of robots. Estimating the pose of objects is an active field with important practical implications, and in the last years, some works (Song et al., 2020; Remus et al., 2023) have been published showing a margin of improvement for several aspects.

**FIGURE 1:**
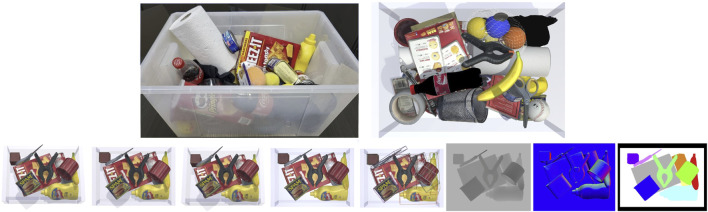
First row: a camera capture of the real cluttered dataset used as base for the virtual dataset generation. Left: an example of a real full cluttered scene; Right: an example of a photorealistic full cluttered scene. Second row: example of the information provided for each scene (from left to right): scene seen by left, middle, and right camera under directional lighting condition; scene seen by middle camera RGB images with spotlighting, and with point lighting also showing the objects bounding boxes; depth image; normal image; segmentation mask.

This work proposes a novel dataset whose peculiarity is the huge amount of clutter in many of the scenes compared to existing datasets regarding the same scope. It provides three different views of the same scene seen under three lighting conditions and seven High Dynamic Range Imaging (HDRI) maps for domain randomization purposes. It is designed to be the companion dataset of the recent benchmark called Cluttered Environment Picking Benchmark (CEPB) (DAvella et al., 2023b), which presents a set of objects selected from existing datasets with the purpose of posing difficulties to the end-effector grasping capabilities and the perception system. Therefore, the dataset is generated synthetically using 3D CAD models of household and industrial objects coming from affirmed benchmarks like YCB (Çalli et al., 2015), ACRV-APC (Leitner et al., 2017; Correll et al., 2018), and TLESS (Hodan et al., 2017) to not reinvent the wheel, and to employ existing objects used in standard manipulation benchmarks. Only very few objects have been introduced, substituting the ones of the original dataset with others having the same characteristics to guarantee easy availability worldwide to buy the physical objects and test real-world scenarios. The scenes are photorealistic images of objects inside a clear box generated through the Unity 3D engine with the support of Flex, a position-based physical simulation library. Thanks to this peculiar physical simulation, the clutters contain both rigid, soft, and deformable objects, and the interaction among the different objects is properly resolved. In addition, by assuming some simplifications, even objects filled with liquid, thus having complex internal dynamics, are considered, resulting in realistic rendering. The level of clutter introduced by the proposed dataset makes it hard to acquire accurate ground truth concerning detection or pose estimation in real environments. Therefore, the choice of synthetic data is justified to overcome such limitations. Figure 1 compares a real bin-picking scene with all the objects of the dataset (on the left) with an example of a photorealistic scene from the top-view central camera (on the right).

Promisingly, the proposed dataset can be helpful for training and testing model-free neural-network-based vision systems for object recognition, detection, pose estimation, or grasping point localization, given the huge number of labeled images. Moreover, it can be usable by model-based approaches since it makes available the 3D CAD models of the objects. In particular, for each scene, the dataset provides the RGB and depth image along with the 2D and 3D object-oriented bounding box (OOBB), a. k.a. cuboid, of the involved objects and the segmentation and normal images, as depicted in Figure 1. Such information is provided in a YAML file that also contains the transformation (translation and rotation) between the camera and the ground truth pose of the objects and the visibility percentage of the objects’ surfaces. Furthermore, nearly 10K separated object images are presented to perform simple tests and compare them with more complex cluttered scenarios tests.

To show the challenges proposed by the presented dataset that should be addressed in the near future to foster an improvement toward autonomous manipulation systems in line with the concept of Industry 4.0, a baseline test has been conducted to evaluate the performance of Deep Object Pose (DOPE) (Tremblay et al., 2018a), which is a state-of-the-art approach based on a neural network. The results show that the performance of the method degrades drastically by introducing even a small amount of clutter in the scene.

The proposed dataset will be distributed publicly, and it will be available on an online repository^1^.

The remainder of the work is organized as follows: Section 2 briefly reviews the most used and recent datasets in the field of vision for manipulation purposes, highlighting the novelty of the proposed dataset; Section 3 introduces the related benchmark, the low-level physical simulation engine, and the network used for the experiments as a baseline to give the proper context for the proposed dataset; Section 4 describes how the dataset is built and its characteristics showing some statistics; Section 5 discusses the results obtained by the DOPE neural network; Section 6 concludes the work.

## 2 Related works

The interest in datasets for object detection, recognition, pose estimation, and grasping pose localization for manipulation purposes has rapidly grown in recent years, especially with the new Industrial Revolution and the advent of deep-learning-based approaches. Good research needs good resources, and deep-learning-based approaches require a large amount of data for good performance. Therefore, in these years, many works made available large databases providing 2D images, point clouds, 3D databases containing clutters and foreground occlusions, and RGB-D datasets for 6D pose estimation or object segmentation (Liu et al., 2021; Selvam Periyasamy et al., 2021).

Hinterstoisser et al. (Hinterstoisser et al., 2013) introduced a dataset regarding object retrieval and pose estimation focusing on 15 textureless objects. It provides about 1200 RGB-D test sequences, including one instance of each object. It is widely adopted but has many limitations, like constant lighting conditions and easily distinguishable uncluttered objects located usually in the center of the image. Later, Brachman et al. (Brachmann et al., 2014) enriched it with annotated occluded objects. The T-LESS (Hodan et al., 2017) dataset introduced 30 textureless industrial-relevant objects with similarities and symmetries in scenes having varying complexity. It provides 39K training and 10K testing images coming from a structured light and a time-of-flight RGB-D camera along with 3D CAD models. ITOOD (Drost et al., 2017) added objects with reflective surface still concerning industrial scenarios. It contains 28 objects organized in 800 scenes acquired by three high-resolution grayscale cameras and two industrial 3D sensors and labeled with 3500 rigid 3D transformations. The BOP (Hodan et al., 2018) benchmark merged the aforementioned dataset, introducing two additional datasets (TUD Light and Toyota Light) concerning other interesting perception aspects, unified their format, and standardized the evaluation procedure. The Rutgers dataset (Rennie et al., 2015) consists of about 10K RGB-D images of hand-annotated 6DOF poses concerning 24 mostly textured objects involved in the cluttered warehouse environment of the Amazon Picking Challenge (APC) held in 2015 (Correll et al., 2018). The dataset also provides the 3D mesh models of the objects. Similarly to the scope of the proposed work, it is aimed to test the perception system during pick and place tasks. However, the Rutgers dataset focuses on picking objects from a shelf while the target of the proposed dataset is bin picking with a higher level of clutter. Very recent works like (Denninger et al., 2019; Morrical et al., 2021) released some general frameworks to help generate photorealistic datasets virtually annotated developed with different programming languages and employing other engines with respect to the ones adopted by the presented work. In addition, they do not provide all the information proposed in this dataset for the purpose of bin picking and do not allow the simulation of all the physical interactions with soft, deformable, and objects containing liquids, which are among the new challenges for robotic grasping and manipulation.

The proposed dataset consists of 50K annotated scenes with at most forty objects per scene providing RGB, depth, normal, and segmentation images with 2D and 3D bounding boxes, translation, and quaternion as ground truth for each of the involved objects, and the objects’ surfaces visibility percentage. The physical simulation reproduces realistic scenarios containing rigid, soft, deformable objects, and objects containing liquids. Furthermore, intense light variation and the use of multiple HDRI maps allow for domain randomization. Table 1 reports the main differences with other existing datasets.

**TABLE 1:** Comparison with existing datasets.

*Dataset*	# Objs	# Frames	Segmentation	Normals	% Visibility	3D poses	Full rotation	Bbox coords	Occlusion	Scene variation	Light variation	Soft and fluid
*Real*												
Linemod (Hinterstoisser et al., 2013)	15	18k	✗	✗	✗	✓	✓	✗	no	no	no	✗
Rutgers APC (Rennie et al., 2015)	24	10k	✗	✗	✗	✓	✗	✗	medium	no	no	✗
T-Less (Hodan et al., 2017)	30	10k	✗	✗	✗	✓	✓	✗	medium	low	no	✗
ITODD (Drost et al., 2017)	28	800	✓	✗	✗	✗	✓	✗	low	no	no	✗
HomebrewedDB (Kaskman et al., 2019)	33	17k	✗	✗	✗	✓	✓	✗	high	small	small	✗
YCB-Video (Xiang et al., 2017)	21	134k	✗	✗	✗	✓	✓	✓	low	medium	low	✗
TUD Light (Hodan et al., 2018)	3	11k	✓	✗	✗	✓	✓	✗	no	high	high	✗
*Synthetic*												
HOPE (Tyree et al., 2022)	28	32k	✓	✗	✓	✓	✓	✓	high	medium	medium	✗
FAT (Tremblay et al., 2018b)	21	60k	✓	✗	✓	✓	✓	✓	small	high	medium	✗
CEPB (ours)	40	50k	✓	✓	✓	✓	✓	✓	extreme	high	extreme	✓

## 3 Background

This section provides some background information to give a proper context for the proposed dataset, introducing the Cluttered Environment Picking Benchmark (CEPB) (DAvella et al., 2023b) that explains some of the design choices behind the dataset generation and structure, the Flex simulation engine used as low-level during the generation process, and the key properties of the DOPE algorithm used as baseline.

### 3.1 CEPB benchmark

**FIGURE 2:**
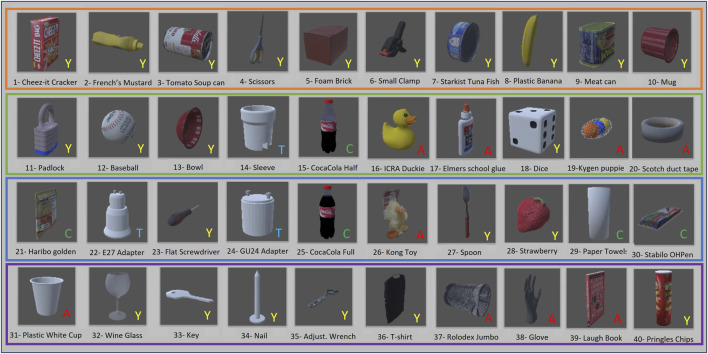
The set of forty objects used in the virtual dataset to generate the cluttered scenes. The objects are separated into four different subsets represented by orange, green, blue, and magenta borders. The original dataset from which the object has been selected is reported as a letter in the right bottom corner with this meaning: A) ACRV, Y) YCB, T) T-LESS, C) CEPB.

The CEPB Benchmark involves the use of 40 objects selected from existing datasets. The characteristics of the items were taken into account during the selection process, evaluating the difficulty that different gripper typologies and vision systems may have in grabbing and perceiving them in cluttered situations, respectively. The protocol guarantees repeatability and comparability but leaves some degree of randomness to emulate the unpredictability of industrial environments thanks to the evaluation metric that considers the difficulty of the clutter. Therefore, forty objects (see Figure 2) belonging to the YCB dataset, the ACRV picking benchmark from the Amazon Picking Challenge, and the T-LESS dataset have been chosen. They can vary in size, shape, and weight and have diverse surface materials and texture properties. There are objects with reflective, perforated, or symmetric surfaces that are challenging for the vision; others have deformable surfaces or strong orientation constraints and shift their center of mass when manipulated. All these problems are accentuated in the clutter because accurate segmentation and stable grasp are more difficult.

The objects are divided into four subsets of ten items each, with an increasing level of difficulty from Subset 1 to Subset 4. It is worth noticing that the difficulty of each subset is the same for every gripper typology in order to not privilege a gripper over the other depending on the nature of the objects. The difficulties have been assigned through a consensus protocol disseminating questionnaires among several colleagues.

The benchmark has a modular design and is organized in *Stages* that can have *intermediate phases*. In principle, *Stages* are meant to represent an industrial-relevant task, which is identified by the *final phase test*, while the *intermediate phases* of each *Stage* aim at evaluating a specific sub-problem (perception, planning, control) of the manipulation task before getting to the *final phase test*, which puts all the intermediate skills together for different objectives. For example, *Stage1* concerns pick and place of non-sequential objects in a cluttered environment which have been addressed by the work in (DAvella et al., 2023c) as the baseline for the benchmark; *Stage2* deals with pick and place of non-sequential unknown objects in a cluttered environment to evaluate the generalization capabilities of the manipulation system; *Stage3* regards pick and place of sequential objects in a cluttered environment mimicking a possible application of the manipulation system in an industrial environment in which the robot interacts with other devices like *PLCs*, receiving information on which target to pick at each time.

The user can apply for each *Stage* independently and even for a specific intermediate phase using one of the subsets or the full dataset. Therefore, the website has a leaderboard for every component of the *Stages*, separating the results per subset.

### 3.2 Flex physical simulation

Flex (Miles et al., 2014) implements a unified particle representation for all types of objects, including gas, solids, liquids, deformable objects, and clothes, based on a real-time position-based dynamics method (PBD) (Müller et al., 2007). The basic building blocks for all the objects’ types of Flex are particles, which allow reducing the number of collision types to process and avoiding complex algorithms for generating contacts between mesh-based representations. Furthermore, in such a way, the simpler interaction among the particles can be resolved in parallel using the GPUs. The position-based dynamics solves a system of non-linear equality and inequality constraints to find the minimum change in the kinetic energy satisfying the constraints, consistently with the principle of least constraint of Gauss. PBD finds the minimum resolving a sequential quadratic programming problem: i.e., it linearizes the constraints and solves a sequence of locally constrained quadratic minimizations. With respect to traditional force-based dynamics methods that accumulate internal and external forces at the beginning of each time step and relate the forces to the accelerations throughout Newton’s second law, the position-based approach has direct control over the positions of objects or the vertices of the meshes without the need of integrations, avoiding overshooting and energy gain problems. Therefore, PBD gains stability, robustness, and speed, keeping the visual results plausible, with the only limitation being that the input mesh should be a manifold. For a detailed overview of Flex and PBD, the authors kindly advise checking the reference papers.

### 3.3 Deep Object Pose

Deep Object Pose (DOPE) (Tremblay et al., 2018a) is a model-based approach that only uses an RGB image as input. First, it estimates the belief maps of 2D keypoints of all the objects in the image coordinate system and then the 6D pose of each object instance with a standard perspective-n-point (PnP) algorithm on the peaks extracted from these belief maps. The final step uses the detected projected vertices of the bounding box, the camera intrinsic parameters, and the object dimensions to recover the final translation and rotation of the object with respect to the camera. All detected projected vertices are used as long as at least four vertices of the cuboid are detected. The network can be trained on synthetic and photo-realistic data without the need for handcrafted labels on real data and provides satisfactory results on real objects thanks to the domain randomization technique.

## 4 CEPB dataset

The dataset uses the objects’ model to render virtual synthetic scenes in Unity 3D, employing the Nvidia Flex engine for physics simulation for each subset of the CEPB Benchmark. Whenever the model of the object was not available or quite noisy in its original dataset, a custom 3D model was generated. The objects were modeled and textured in Blender, and for simple geometric shapes, the combination of CAD modeling and texturing gives results resembling the quality of more involved techniques (Narayan et al., 2015). Furthermore, when not aligned, translations and rotations were applied to center the coordinate frame at the object’s centroid and to align the coordinate axis with those of the objects.

**FIGURE 3:**
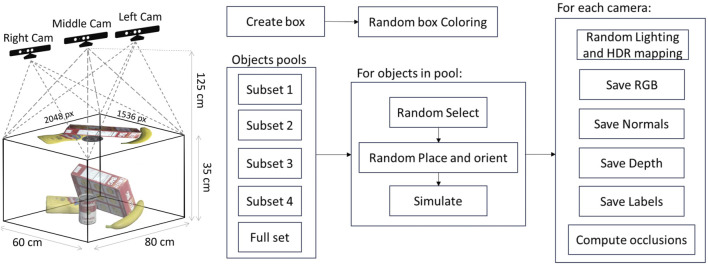
Left: setup of the virtual scene. The middle RGB-D camera is pointing down from a 1.25 m height. The visible workspace is contained in an 80 × 60 × 35 cm volume that is rendered on a 2048 × 1536 pixels image. The other two cameras are shifted on the sides of the middle camera by 3 cm each to obtain a stereo pair with a binocular distance similar to the human eyes. The Left and Right cameras are also shifted on the back and slightly tilted to obtain a visual that is not perpendicular to the working table, still looking at the center of the bin. Right: pipeline of the dataset creation. A clear box is generated over a table. Objects are randomly spawned over the box and left to fall inside it. Physical simulation is performed with the Flex engine to simulate the interaction and collisions within the objects. For each of the cameras, a set of images is generated with varying lighting and mapping.

For each subset, 300K images (RGB, depth, normal, and segmentation) are generated, for a total of 1500K set of images, including 300K scenes containing the overall dataset of 40 objects. Each scene, seen by three cameras, is subject to three lighting conditions (directional light, a point light, and a spotlight) and seven different HDRI maps used for domain randomization purposes. A second randomization step is performed on half the dataset concerning the color, intensity, and direction variation of the scene lights and the bin color. For the four subsets, the workspace consists of a 292*mm* × 429*mm* × 149*mm* clear box (the one used by the YCB dataset), while for the overall set of objects, the clear box is of 390*mm* × 560*mm* × 280*mm* size (standard IKEA box). In both cases, one virtual camera has been placed at a 1.25 *m* height above the workspace level, looking downward at the workspace containing the cluster of objects, and the other two are rotated with +15° and −15° respect to the middle one, looking at the center of the bin (see Figure 3).

A schematic of the elements in the dataset generation pipeline is reported in Figure 3. In each run, every 2 s, one item at a time in the specific set falls in the clear box starting at the height of 25 cm from the bin with a random position that allows the object to always fall inside the clear box. The random spawn and the generation rate avoid the intersection of the objects at the starting time. Then, they can collide with each other, thanks to the Flex physics engine. Therefore, the generation time is about 30 s per scene for each subset (about 3 s per object) using an NVIDIA GeForce RTX 3090 graphic card. For every scenario, the following information is provided for each object:

• 3D object-oriented bounding box in world-space and screen-space coordinates as a list of eight points enclosing the objects in the scene;
• world (camera) coordinates as a translation vector (3D centroid) and a quaternion;
• 2D centroid in screen-space coordinates;
• visibility percentage, computed as the number of visible pixels over the whole object projected space in its pose. This value is obtained during the rendering stage taking advantage of a custom compute shader that can access fragment data.

The RGB and depth images are provided in 2K resolution along with the intrinsic camera parameters. Figure 1 shows an example of a subset scene seen by the three different points of view and under the three different lighting conditions and the normals and segmentation images.

**FIGURE 4:**
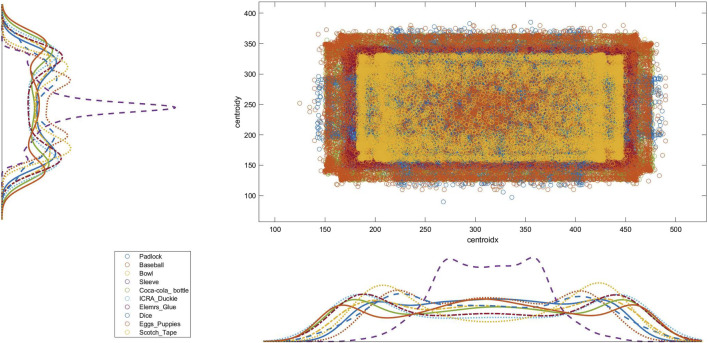
Distribution of each object in Subset two showing the *x* − *y* centroid coordinates.

**FIGURE 5:**
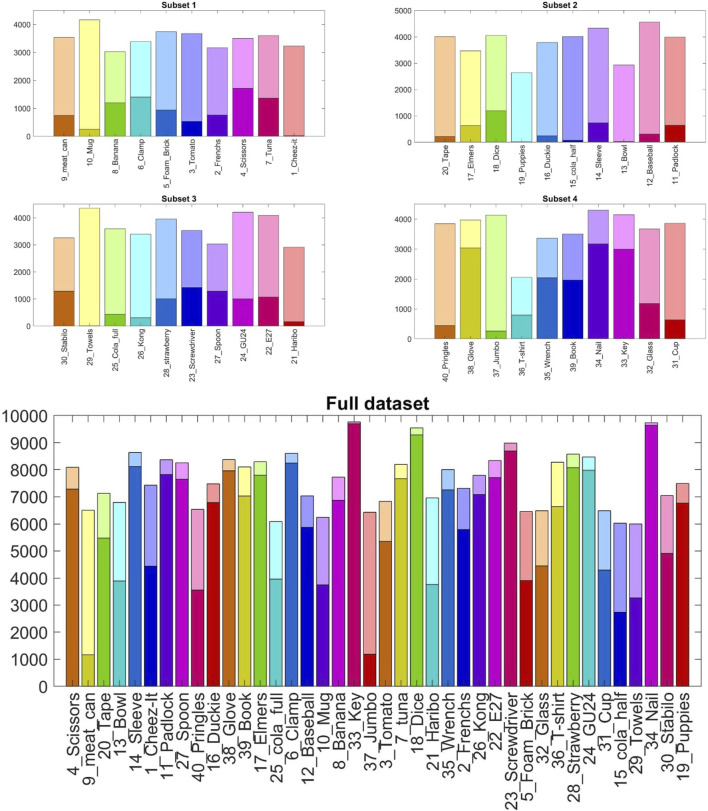
Visibility of the forty objects in each subset and in the full dataset over 10K frame each. Light color bars indicate highly visible objects (occlusion less than 25% of the item’s volume), while solid color bars represent highly occluded objects (occlusion more than 75% of the item’s volume).

In each subset, the statistical distribution of the pose covered by every object follows an almost uniform distribution profile over the total amount of generated frames. Figure 4 depicts the distribution of Subset two considering the *x* − *y* centroids of each object. The objects’ distribution mainly differs due to their relative shape and size, and the interaction constraints with the enclosing box. The four subsets put the objects in the smaller clear box, while the full dataset uses the larger one to cope with the different amounts of clutter volume. Figure 5 gives an insight into the different levels of difficulty needed to identify each object in the various scenarios due to the diverse clutter percentage. In particular, the solid color bars represent the number of frames in which the specific object is visible for less than 25% of its volume (highly occluded), while lighter color bars show the number of occurrences in which the object is visible for more than 75% (highly visible). The visibility of each object has been computed by a custom fragment shader pass during the rendering phase of dataset generation.

**FIGURE 6:**
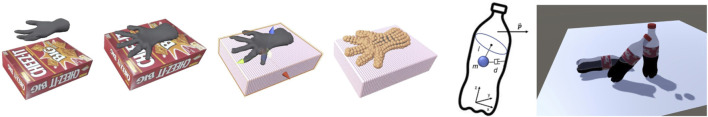
Left: interaction between a glove and a Cheez-it cracker box. When the glove falls over the box its shape is deformed. The last two images on the right show the particle representation of the cracker box and the deformed glove state. Right: liquid dynamic simulation. The first image is the representation of the sloshing dynamics. The second and last one is the results of the sloshing dynamic with the developed shader.

Thanks to the flexibility of the method, the dataset presents cluttered scenarios that mix rigid, soft (like “Kong Toy”), and deformable (like “Glove” or “t-shirt”) objects. Figure 6 (left) shows the visual and physical representations of two objects during a collision interaction. From a technical point of view, the simulation of a stable interaction within multiple objects is not straightforward, and considering the coexistence of rigid, soft, deformable objects, and fluids, the proposed dataset is unique. Even in a PBD physical simulation, when a large number of objects share the same environment in a cluttered scenario, there is the need to simplify objects’ shapes and limit the number of collision particles to properly solve the physical iteration steps. In particular, in the development of the dataset, all the object meshes have been optimized to improve the physical computation capability. In some cases, a novel geometrical shape has been designed to reduce the physical interaction efforts.

In order to simulate objects filled with liquid, thus presenting complex internal dynamics, like the plastic bottles involved in the dataset, a single, damped pendulum (Jan et al., 2019; Gattringer et al., 2022) with length *l*, mass *m*, damping constant *d*, and translational acceleration $\ddot{p}$ has been implemented directly during object rendering with a custom shader. Such a solution can simplify the computational complexity of Flex in handling confined liquids and provide a more satisfying visual rendering. In particular, a fragment shader is responsible for the visual representation of the liquid inside the bottle according to the object’s motion, and a shadow caster pass generates corresponding shadows and depth maps. The result can be appreciated on the right of Figure 6.

## 5 Experiments and discussion

The DOPE neural network has been used to assess the difficulties of the proposed dataset. In particular, the experiments have been performed against subset 1, using the point of view of the central camera under the directional lighting condition.

Table 2 reports the results of the DOPE neural network on the same subset concerning the following metrics:

• *n_detected* is the number of objects detected in the various frames. It can be larger than 5000 (the number of frames) since the baseline may look for false positive detection, recognizing twice the same object.
• *n_depth error* is the number of times the baseline wrongly estimated the depth of the object, going beyond the depth of the box.
• *low visibility* is when the visible surface percentage of the items is less than 10% of the total surface, making the pose estimation very difficult. Therefore, depending on the dimension of each object and the object’s arrangement, such a value can be very different. Roughly speaking, it is more likely that small objects may have higher low visibility metrics.
• *Visible Surface Discrepancy (vsd)* gives the average of pixels belonging to the estimated and ground-truth visibility masks of the distance maps obtained by rendering the object model in the estimated and ground-truth pose that are under a given threshold. It is worth noticing that VSD treats indistinguishable poses as equivalent by considering only the visible object part. It can be seen as an extension of the Complement over Union (cou), which is the cost function related to the popular Intersection over Union score used for measuring the accuracy of detection and segmentation methods in the 2D domain. The fraction of annotated object instances, for which a correct pose is estimated, is referred to as recall, and for the experiments, AR_vsd is the average of recall rates obtained ranging the misalignment tolerance from 5% to 50% of the object diameter with a step of 5% and the threshold of correctness ranging from 0.05 to 0.5 with a step of 0.05.
• *Maximum Symmetry-Aware Surface Distance (mssd)* provides the maximum distance between the model vertices taking into account the set of global symmetry transformations. Such a metric is relevant for robotic manipulation, where the maximum surface deviation strongly indicates the chance of a successful grasp. For the experiments, AR_mssd is the recall of maximum symmetry-aware surface distance error.
• *Maximum Symmetry-Aware Projection Distance (mspd)* is the 2D equivalent of the previous metric using the 2D projection function. Compared to the 2D projection (proj), such a metric considers global object symmetries and adopts the maximum distance in favor of the average in order to increase its robustness against the sampling of mesh vertices. For this reason, the alignment along the optical (Z) axis is not considered, and the metric measures only the perceivable discrepancy. It is relevant for augmented reality applications and suitable for evaluating RGB-only methods, for which estimating the alignment along the optical axis is more challenging. For the experiments, AR_mspd is the recall of maximum symmetry-aware projection distance error.

**TABLE 2:** DOPE results: the values reported in the table are an average among all the 5K frames for each object.

Object	n detected	n depth error	n low visibility	AR_vsd	AR_mssd	AR_mspd	
Cracker	4,954	206	549	0.70	0.78	0.83	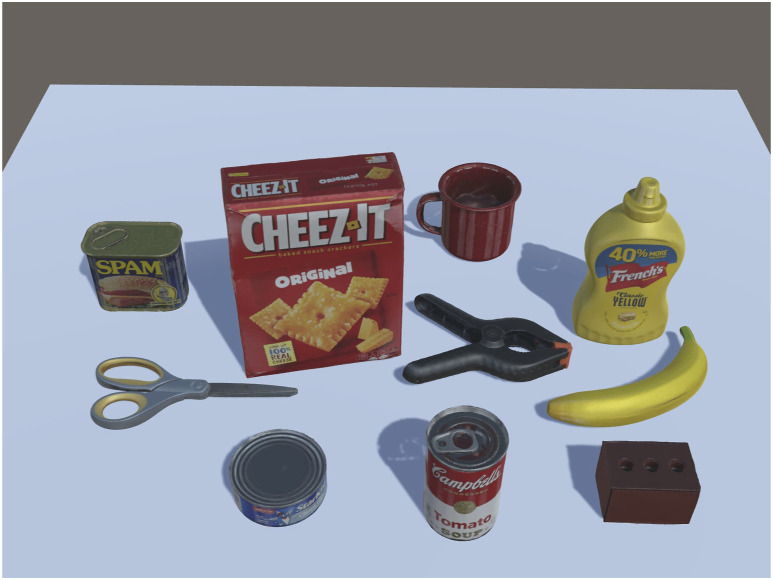
Tuna	593	239	2036	0.06	0.02	0.09	
Mustard	4,035	576	1698	0.41	0.52	0.71	
Tomato Soup	5363	1245	1056	0.25	0.40	0.66	
Banana	1984	961	2,319	0.15	0.10	0.22	
Scissors	1366	464	2,470	0.10	0.08	0.24	
Clamp	1902	553	2,257	0.17	0.07	0.15	
Mug	286	156	565	0.08	0.01	0.03	
Foam	829	568	1514	0.03	0.01	0.06	
Meat Can	4,101	997	1367	0.29	0.37	0.71	

The Cheez-it cracker box obtained the best performance on all the metrics and was not detected only in 64 scenes even though the object had low visibility, i.e., less than 10%, in 549 scenes. However, the estimations of the network were out of the feasible depth limit in 206 scenes. Similarly, the mustard bottle got good performance compared to all the other items. The results can be explained by the fact that the Cheez-it cracker box and the mustard bottle are the biggest objects in the scenes with a good texture to be recognized even in case of medium occlusions. For all the other objects, the number of detections decreased significantly, and for the detected scenes, the *svd* metrics are greater than 0.5 with a high percentage of out-of-feasible depth range estimations compared to the low visibility conditions. It is worth noticing that even if the meat-can has a good detection rate, the *vsd* metric is poor, along with a great depth range error. In addition, the tomato soup has been detected more times than required contributing to lowering its performance, as the number of detections is greater than the total number of scenes.

### 5.1 Progressive clutter study

To better understand the obtained results, a study collecting the AR_vsd metric for progressive scenes presenting one to ten objects is conducted. In particular, 100 images for each number of objects in the scene are used, and the metric is then averaged. Table 3 depicts the degradation of performance, showing that DOPE can handle a small amount of clutter, working quite well up to three objects, and then starts failing or becoming unprecise due to increasing clutter.

**TABLE 3:** Progressive Clutter Study: DOPE performance on progressive scenes containing from 1 to 10 objects.

# Object	1	2	3	4	5	6	7	8	9	10
AR_vsd	0.82	0.74	0.42	0.37	0.31	0.27	0.25	0.24	0.23	0.22
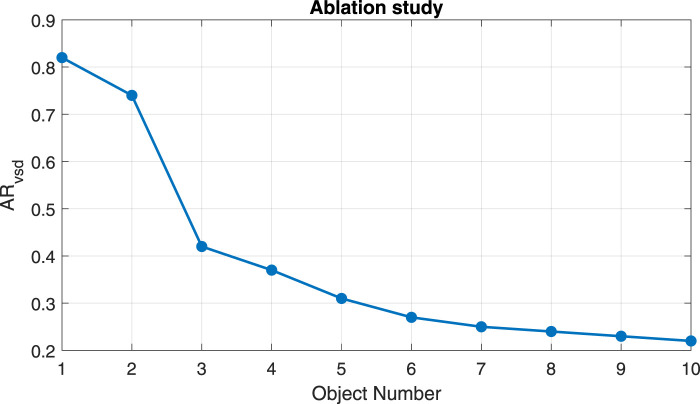										

## 6 Conclusion

This paper presents the companion dataset of the CEPB benchmark for bin-picking in heavily cluttered scenes. The aim of the dataset is to provide massive and rich data for training object detection, pose estimation, or grasping neural networks for the future challenges required by the industrial 4.0 and 5.0 paradigms. In particular, it provides about 1500K of virtually generated photo-realistic images of 50K annotated cluttered scenes mixing rigid, soft, and deformable objects of varying sizes under three different points of view and three light conditions and seven HDRI maps for domain randomization purposes. The objective of the dataset was to provide a substantial but manageable size of multiple modalities of photorealistic and fully annotated frames presenting different levels of clutter difficulties, and that would be future-proof, i.e., that will be solved in the next future but not solved by the present state-of-the-art techniques. Therefore, a baseline with the DOPE neural network has been provided using the objects belonging to subset 1, showing the degradation of the performance of this state-of-the-art method passing from a single object scenario to a clutter with 10 objects.

Since the experiments showed a degradation of the performance of a current state-of-the-art network related to the amount of clutter proposed by the CEPB dataset, it would be interesting in future work to train such a network with the proposed dataset to let the network understand during training how to handle such complex scenarios. Then, the next step would be to conduct a more in-depth analysis of the performance of the DOPE network and other 6D pose estimation networks exploiting different principles at different levels of clutter before and after the training on the CEPB dataset to measure the boost in performance the proposed dataset may enable for future robotic grasping applications.

## Data Availability

The original contributions presented in the study are included in the article/Supplementary materials, further inquiries can be directed to the corresponding author.

## Footnotes

1 at http://cepbbenchmark.eu
